# Xylanase Inhibitors: Defense Players in Plant Immunity with Implications in Agro-Industrial Processing

**DOI:** 10.3390/ijms232314994

**Published:** 2022-11-30

**Authors:** Silvio Tundo, Giulia Mandalà, Luca Sella, Francesco Favaron, Renesh Bedre, Raviraj M. Kalunke

**Affiliations:** 1 Department of Land, Environment, Agriculture, and Forestry (TESAF), University of Padova, 35020 Legnaro, Italy; 2 Department of Biotechnology, University of Verona, Strada Le Grazie 15, 37134 Verona, Italy; 3 Texas A&M AgriLife Research and Extension Center, Texas A&M University System, Weslaco, TX 78596, USA; 4 Donald Danforth Plant Science Center, 975 N Warson Rd, 7 Olivette, St. Louis, MO 63132, USA

**Keywords:** hemicellulose, xylanases, cell wall degrading enzymes (CWDEs), plant defense, cell death, food processing, allergenicity

## Abstract

Xylanase inhibitors (XIs) are plant cell wall proteins largely distributed in monocots that inhibit the hemicellulose degrading activity of microbial xylanases. XIs have been classified into three classes with different structures and inhibition specificities, namely *Triticum aestivum* xylanase inhibitors (TAXI), xylanase inhibitor proteins (XIP), and thaumatin-like xylanase inhibitors (TLXI). Their involvement in plant defense has been established by several reports. Additionally, these inhibitors have considerable economic relevance because they interfere with the activity of xylanases applied in several agro-industrial processes. Previous reviews highlighted the structural and biochemical properties of XIs and hypothesized their role in plant defense. Here, we aimed to update the information on the genomic organization of *XI* encoding genes, the inhibition properties of XIs against microbial xylanases, and the structural properties of xylanase-XI interaction. We also deepened the knowledge of XI regulation mechanisms *in planta* and their involvement in plant defense. Finally, we reported the recently studied strategies to reduce the negative impact of XIs in agro-industrial processes and mentioned their allergenicity potential.

## 1. Introduction

The plant cell wall is a complex and metabolically active matrix surrounding plant cells. In plant–microbe interactions, the plant cell wall plays several important roles. It represents the main pre-formed physical barrier that pathogens must overcome to penetrate and colonize the host tissue, a source of nutrients for invaders, and its partial degradation can produce signaling molecules acting as damage-associated molecular patterns (DAMPS) [1]. The plant cell wall is mainly composed of polysaccharides such as cellulose, xyloglucan, β-1,3:1,4-glucan, xylan, mannan, pectin, and callose [2]. Other components are phenolic compounds, proteins, and water. Notably, callose and phenolic compounds are deposited in response to pathogen attacks to reinforce the cell wall [3]. Cells that have completed cellular expansion can generate a secondary cell wall composed of cellulose, hemicellulose, and lignin. Lignin deposition is both developmentally and stress-induced and represents a barrier against pathogen colonization [4].

To overcome the plant cell wall, most pathogens, during pathogenesis, as well as many saprophytic microbes, secrete a wide array of cell wall-degrading enzymes (CWDEs) with different substrate specificity, which provide a source of nutrients and can facilitate the penetration and/or colonization of the microorganisms into the host tissues. Microbial CWDEs are also produced in biofactories and have applications in several industrial processes [5]. *CWDEs* are represented in multigene families that share sequence and structural similarities and that, in some cases, have redundant functions [6]. These enzymes can cleave glycosidic bonds accounting for the rapid and extensive degradation of plant cell wall polysaccharides [7]. The genomic organization of *CWDEs* has been investigated in phytopathogenic fungi, oomycetes, and nematodes [8,9,10].

Among CWDEs, xylanases are key enzymes in hydrolyzing the β-1,4 linkages of xylan, the main component of hemicellulose [11].

Hemicelluloses are a group of complex polymers composed of different hexose, pentose, and/or uronic acid sugars and have different structures [12]. In the primary cell wall of graminaceous monocot plants, the most abundant hemicellulose is arabinoxylan, a polysaccharide composed of a backbone of β-1,4-D-xylose substituted with arabinose and, less frequently, with glucuronic acid, methylglucuronic acid, and acetyl side groups in a non-repeating fashion [13]. The most abundant hemicellulose in the dicot primary cell wall is xyloglucan, a linear D-glucose backbone linked by β-1,4-bridges substituted at regular sites with D-xylosyl residues [14] and further extended by galactosyl, fucosyl, or arabinosyl residues [15]. Overall, the degree of substitutions and the side chains vary considerably between plant species and cell types [13].

The network of cellulose and hemicellulose is embedded in a matrix of pectin, resulting in a strictly interwoven network of cross-linked fibers [16].

Complete hydrolysis of hemicellulose requires the action of several enzymes. The carbohydrate-active enzymes (CAZy) database (www.cazy.org, accessed on 1 September 2022) classifies the microbial CWDEs in glycoside hydrolases (GH) families. According to the sequence-based classification of the CAZy database, the xylanases have been classified in the GH families 3, 5, 7, 8, 10, 11, 30, 39, 43, 52, and 54 [17] and comprise a catalytic domain with a demonstrated endo-β-1,4-xylanase activity. Nevertheless, most xylanases have been grouped into families GH10 and GH11, corresponding, respectively, to high (>30 kDa) and low (<30 kDa) molecular weight xylanases.

The degradation of xylan contained in the plant cell wall is one of the mechanisms potentially involved in the pathogenesis of fungal pathogens [6]. However, xylanases contribute to pathogenesis also with their capacity to induce cell death in the host tissues independently of their enzymatic activity [18,19].

However, plants are not defenseless. Indeed, they evolved xylanase inhibitors (XIs) as a mechanism of disease resistance. This hypothesis is supported by the observation that XIs inhibit the activity of microbial but not plant-derived xylanases and by studies in which the expression *in planta* of XIs confers increased resistance against fungal pathogens such as *Fusarium graminearum*, *Botrytis cinerea*, *Pyricularia oryzae* and herbivores [20,21,22,23]. The increased resistance against *F. graminearum* is a common trait shared with plants expressing other CWDEs inhibitors, such as polygalacturonase inhibiting proteins (PGIP) and pectin methylesterase inhibitors (PMEI) [24,25,26,27]. The first reports demonstrating the presence of XIs date back to the 1990s, when xylanase inhibition activity was found in wheat extracts [28]. Soon after, the same research group isolated and characterized the first XI, the *Triticum aestivum* xylanase inhibitor (TAXI) [29]. Another proteinaceous inhibitor of xylanases was assigned to a new class of XI, namely xylanase inhibitor protein (XIP) [30]. Although the N-terminal sequence of XIP showed 86% identity with the sequence of chitinase III from rice, the inhibitor did not show chitinase activity. The last class of XIs identified in wheat is the thaumatin-like xylanase inhibitor class (TLXI) [31] which shows >60% similarity to thaumatin-like proteins (TLPs) and for which a role in plant disease resistance has been reported [32].

A complete and detailed review by Dornez et al. (2010) [33] provides an overview of the genomic organization of *XI*-encoding genes in wheat, the biochemical and structural properties of XIs, and their involvement in plant defense. In this review, we analyze the distribution and genomic organization of *XI*-encoding genes of three other cereal plants (sorghum maize and rice), summarize the research on the inhibition specificities of monocot XIs against microbe xylanases, and update the molecular features of xylanase–XI interactions. We also analyze the regulatory mechanisms of XIs in planta and following pathogens’ attack, and we focus on the role of XIs in plant defense with particular attention to the mechanisms underlying resistance. Since the presence of XIs in food matrices decreases the efficiency of xylanases used in agro-industrial processes, we report the strategies to reduce the negative impact of XIs on food processing and quality. Finally, we address the issue regarding the involvement of XIs in adverse reactions such as food allergies.

## 2. XIs Genomic Organization

Genomic and comparative studies of gene clusters help us determine the degree of conservation, divergence, and functional correlation and provide clues to their evolutionary footprints. Recent advances in DNA sequencing technology, combined with decreasing sequencing costs, have resulted in an explosion in the quantity and quality of publicly available plant genome sequences, providing unprecedented opportunities for comparative genomic studies in plants [34]. Our previous research into the genomic organization and distribution of *PGIP* genes in legumes suggested that the evolutionary forces driving the *PGIP* genes follow a birth-and-death model [35].

Due to the predominance of xyloglucan in the hemicellulose of dicots and non-graminaceous monocot cell wall, xyloglucan-specific endoglucanases inhibitor protein (XEGIP) genes with homology to TAXI-type endoxylanase inhibitors from monocots have been found in their genomes [13,36,37]. Conversely, *XI* genes have typically evolved in genomes of graminaceous species, characterized by arabinoxylan-rich hemicellulose [13]. The differences in cell wall composition between monocots and dicots could explain the evolution and the functional diversification of XIs in monocots rather than dicots.

In this review, we present a comprehensive overview and an update about the genomic organization of XIs (TAXI and XIP) across genomes of important monocot crop plants such as rice, maize and sorghum to understand the genomic distribution, conservation, and evolutionary driving forces.

The publicly available protein sequences of sorghum, maize, and rice were downloaded from the Phytozome database (https://phytozome-next.jgi.doe.gov/, accessed on 1 September 2022). The conserved protein domains were predicted in protein sequences by HMMER software (http://hmmer.org/, version 3.3) using protein family hidden Markov model (HMM) profiles (Pfam) [38]. The presence of the signature protein domains TAXI (for *TAXI* genes), Glyco_hydro_18, and GH18_hevamine_XipI_class_III (for *XIP* genes) was used for TAXI and XIP family classifications. These signature domains are based on TAXI-I (AJ438880.1) and XIP-I (ADB81849.1) for the respective families. Phylogenetic analysis was used to further validate the identified TAXI and XIP family members from sorghum (Supplementary Figure S1), maize (Supplementary Figure S2), and rice (Supplementary Figure S3). We included well-characterized wheat TAXI sequences in the phylogenetic analysis of the TAXI family members: TAXI-IA (CAD27730.1), TAXI-IIA (CAG26970.1), TAXI-IB (CAG26972.1), TAXI-IIB (CAG26971.1), TAXI-III (BAD72883.1), and TAXI-IV (BAD72882.1). Wheat TAXI sequences, as expected, shared a considerable degree of similarity with the identified sorghum, maize, and rice TAXI family members.

The XIP sequences share a high similarity with chitinases; therefore, we used chitinases from respective crop species (sorghum chitinase: Sb01g021920.1 CHI; maize chitinases: GRMZM2G057093 P01 CHI, GRMZM2G162505 P01 CHI; rice chitinases: BAA22266.1 OsChib3b, BAA21743.2 OsChib3a) in the phylogenetic analysis of the XIP family members. Chitinases from the respective crop species (sorghum, maize, and rice) were separated from identified XIP family members (Supplementary Figures S1–S3).

We also attempted to identify TLXI family members in these crops, but the high homology of TLXI with thaumatin-like proteins (TLP) does not allow an accurate TLXI family member identification.

*TAXI*-encoding genes have been well characterized in a recent genome-wide identification study, including chromosomal localization in wheat [39]. The authors found a total of 277 *TAXI* genes, which were located on 21 chromosomes of *Triticum aestivum* L. and divided into 6 subfamilies. The *TAXI* genes belonging to the same subfamily showed a similar organization of introns and exons in the gene sequence and shared the same conserved protein motifs. The high number of tandem replication and segmentally duplicated genes found in wheat suggests that tandem and segmental duplications could be the main source of new *TAXI* genes in wheat, together with polyploidization. It is noteworthy that several *TAXI* genes from wheat, barley, and rye have been isolated and cloned [21,33,39].

**Figure 1:**
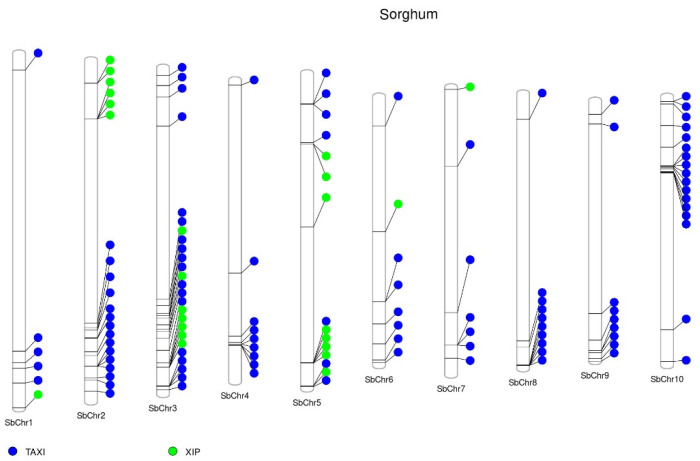
Distribution of xylanase inhibitors (XIs) in sorghum (*Sorghum bicolor*) chromosomes. The publicly available protein sequences of sorghum were downloaded from the Phytozome database (https://phytozome-next.jgi.doe.gov/, accessed on 1 September 2022). The conserved protein domains of the XIs were predicted in protein sequences by HMMER software (http://hmmer.org/) using a protein family hidden Markov model (HMM) profile (Pfam) database [38]. SbChr, chromosome. Web-based interface PhenoGram was used to annotate the genes’ location on the chromosome [40].

The available sequences deposited in the Phytozome database highlight the presence of 103 *TAXI* genes in the sorghum genome. Chromosomes 2, 3, and 10 contain the highest number of *TAXI* genes: 15, 18, and 17 genes, respectively, accounting for around half (50 genes) of all *TAXI* genes. One cluster with seven *TAXI* genes was found on chromosome 8 (Figure 1, Supplementary Figure S4).

**Figure 2:**
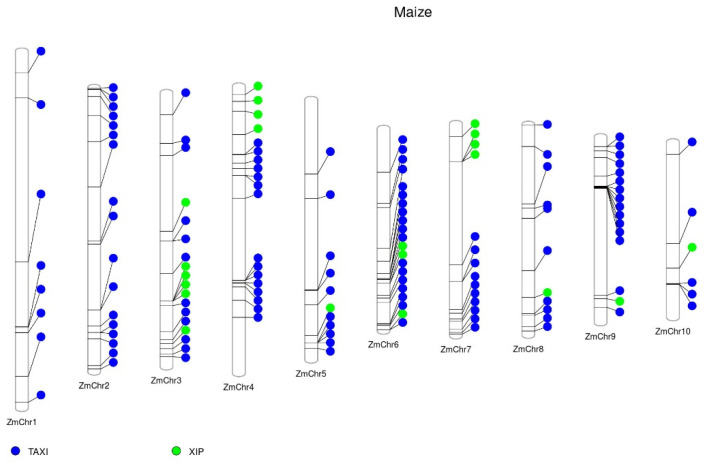
Distribution of xylanase inhibitors (XIs) in maize (*Zea mays*) chromosomes. The publicly available protein sequences of maize were downloaded from the Phytozome database (https://phytozome-next.jgi.doe.gov/, accessed on 1 September 2022). The conserved protein domains of the XIs were predicted in protein sequences by HMMER software (http://hmmer.org/) using a protein family hidden Markov model (HMM) profile (Pfam) database [38]. ZmChr, chromosome. Web-based interface PhenoGram was used to annotate the genes’ location on the chromosome [40].

The maize genome has 120 *TAXI* genes, and the cluster with the highest number of genes (8) was found on chromosome 9 (Figure 2, Supplementary Figure S5). Most *TAXI* genes are found on 4 chromosomes: on chromosome 2 (17 *TAXI* genes), chromosome 4 (15 *TAXI* genes), chromosome 6 (18 *TAXI* genes), and chromosome 9 (15 *TAXI* genes), accounting for more than half (65 genes) of all the *TAXI* genes (Figure 2, Supplementary Figure S5).

**Figure 3:**
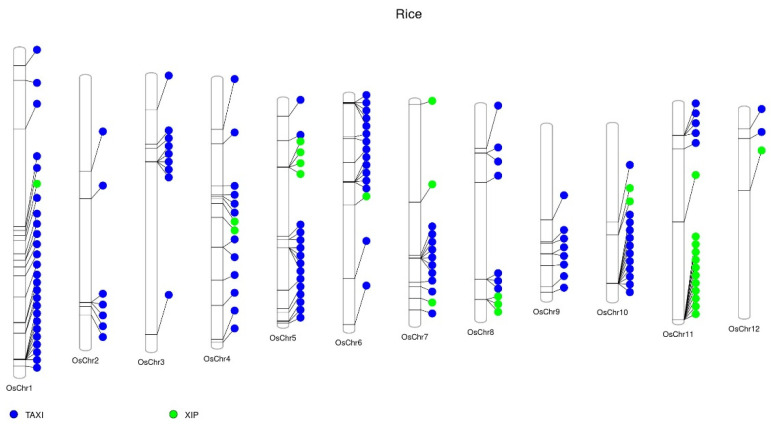
Distribution of xylanase inhibitors (XIs) in rice (*Oryza sativa*) chromosomes. The publicly available protein sequences of rice were downloaded from the Phytozome database (https://phytozome-next.jgi.doe.gov/, accessed on 1 September 2022). The conserved protein domains of the XIs were predicted in protein sequences by HMMER software (http://hmmer.org/) using a protein family hidden Markov model (HMM) profile (Pfam) database [38]. OsChr, chromosome. Web-based interface PhenoGram was used to annotate the genes’ location on the chromosome [40].

The rice genome contains 127 *TAXI* genes. Chromosome 1 has the highest number of *TAXI* genes (25 genes), followed by chromosomes 5 and 6, which have both 15 *TAXI* genes, and chromosome 10 harboring 12 *TAXI* genes (Figure 3, Supplementary Figure S6). These 4 chromosomes (1, 5, 6, and 10) account for more than half (67 genes) of all the rice *TAXI* genes. Interestingly, one cluster on chromosome 10 contains 11 *TAXI* genes arranged in tandem repeats, which is the highest number of *TAXI* genes in one cluster so far observed. Each of the sorghum, maize, and rice genomes has at least one cluster of 4–11 *TAXI* genes (Supplementary Figures S4–S6).

More than 100 complete or partial *XIP* genes from monocot plants have been deposited in nucleotide databases (http://www.ncbi.nlm.nih.gov/, accessed on 1 September 2022) based on BLASTp search using wheat XIP-I (ADB81849.1) as a query. In wheat, among XIP type XIs, *XIP-I*, *XIP-II*, *XIP-III*, *XIP-R1*, *XIP-R2*, *xip-9023*, *xip-366*, and the three homologous *XIP-6A*, *XIP-6B,* and *XIP-6D* [33,41,42] have been successfully cloned. Brachypodium, sorghum, maize, and rice genomic data were used to define XIP genomic organization [43]. The study showed that most of these genes are organized in clusters. Our search of databases showed that the sorghum genome contains a total of 24 *XIP* genes, 17 of which are distributed in five clusters on three different chromosomes: one cluster is located on chromosome 3 with five *XIP* genes, whereas on chromosomes 2 and 5 are located two clusters with four and two genes (Figure 1, Supplementary Figure S7).

The maize genome contains 21 *XIP* genes (Figure 2, Supplementary Figure S8). Among them, nine genes are distributed in three clusters on three different chromosomes. One cluster with four genes is located on chromosome 3, and one cluster located on chromosome 7 has three genes, whereas chromosome 6 contains one cluster with two genes.

The rice genome contains 29 *XIP* genes; it has four *XIP* gene clusters (Figure 3, Supplementary Figure S9) distributed over four different chromosomes. Interestingly, on chromosome 11, it is located a cluster with 11 *XIP* genes arranged in tandem repeats.

Based on the above-mentioned gene clustered genomic organization, it was speculated that the cluster organization of *XIP* genes could be associated with horizontal gene transfer from fungal genomes [43] given the high similarity of *XIP-I* genes from wheat with the fungal chitinase genes [44,45]. This suggests the possibility of horizontal gene transfer followed by “cut-and-paste” transposons associated mechanism, chromosomal translocations, genome shuffling, uneven crossing-over followed by tandem duplications or co-evolution. However, more comparative genomic organization studies are required to confirm this hypothesis [43].

To complete our analysis, we performed a BLAST search on NCBI using TAXI-I (AJ438880.1) and XIP-I (ADB81849.1) with the aim of finding matches with dicot XIs (Supplementary Figure S10). However, we did not find any match. These results account for the functional diversification of XIs in monocots rather than dicots.

Although there is a high number of *XI*-encoding genes in monocots, inhibitory activity has only been demonstrated for a few of them. As previously reported for *PGIP* genes, *XI*-encoding genes also show both functional redundancy and diversification [46]. The evolution of new XIs could have an adaptative significance to stress-related conditions and reflects the high functional variability of xylanases secreted by phytopathogens. The genomic analyses highlight a higher number of *TAXI* genes as compared to *XIP*. Noteworthy, the high similarity of *XIP* with chitinases and of *TLXI* with *TLP* would suggest a different evolutionary path as compared to *TAXI* gene families with the possibility of acquiring the capacity of inhibiting xylanases as an additional function. Notably, similarly to XIP and TLXI, TLPs and chitinases play an essential role in plant defense [47,48]. Therefore, the origin of *XIP* and *TLXI* could be associated with functional divergence from members of *TLP* and chitinase gene families, respectively.

## 3. Inhibition Activity of XIs

The interaction between the different classes of XIs and microbial xylanases is characterized by high specificity. Indeed, TAXI-type and TLXI-type inhibitors inhibit GH11 but not GH10 xylanases [31,49]. Differently, XIP-type inhibitors inhibit the xylanases belonging to both GH10 and GH11 families, although there is high variability in the inhibition efficacy towards xylanases belonging to the GH11 family. Remarkably, XIP-type inhibitors inhibit fungal but not bacterial xylanases [44]. However, a recent study demonstrates the capacity of XIP-I to inhibit xylanase of the rumen bacterium *Paenibacillus* sp. [41]. Further investigations would be helpful in unraveling the molecular mechanism underlying that inhibition.

The levels of TAXI and XIP-type inhibitory activities vary considerably in cereals; they are higher in bread wheat than in barley and oat [50]. Higher inhibitory activity was detected in bread wheat than in durum wheat [51]. No significant differences were found among the common wheat cultivars tested. The same authors detected comparable inhibitory activity in barley and rye. TAXI and XIP inhibitory activities are higher in bran than in flour, as demonstrated in hard spring wheat cultivars, providing evidence for the higher concentration of these inhibitors in the grain’s outer layers and for a role in plant defense. However, levels of XIP-type inhibitors in hard spring wheat are about three to eight times higher as compared to TAXI-type inhibitors. The variability in the levels of TAXI in wheat is explained mainly by genotype, indicating that TAXI inhibitory activity is under genetic control, while the genotype contribution to variability is lower for XIP [52].

The inhibition kinetics of TAXI and XIP inhibitors from wheat and rice against the enzymatic activity of fungal xylanases revealed that the mechanism underlying the inhibition is competitive, suggesting that TAXI and XIP inhibitor proteins bind to the active site, thus preventing the substrate from binding to the active site of the enzyme. Considering the *Ki* values from inhibitory activity assays, the range of inhibition of XIP-I against fungal xylanases is quite extensive, varying from 2.1 nM against *B. cinerea* BcXyn11a to 610 nM against the *Trichoderma viride* TvXyl (Table 1). Differently, TAXI-type inhibitors have a narrower range of inhibition since *Ki* values vary from 2.59 nM of TAXI-III against *F. graminearum* FGSG_03624 to 46 nM of TAXI-I and TAXI-II against *Penicillium funiculosum* PfXynA (Table 1).

Unlike TAXI and XIP, TLXI is a non-competitive inhibitor that inhibits GH11 xylanases. However, it is not able to inhibit high pI GH11 xylanases from *Trichoderma longibrachiatum* (Xyn II, pI 9.0) and *Bacillus subtilis* (BsXynA, pI 9.3) [31]. TLXI is a highly stable protein, even at extreme values of pH and temperature, more than observed for TAXI or XIP [80]. Based on *Ki* values from inhibitory assays, the range of inhibition of TLXI inhibitors is narrower as compared to XIP inhibitors but larger as compared to TAXI inhibitors since the lower *Ki* value determined is 4.2 nM against Xyn I xylanase of *T. longibrachiatum* and the higher is 289.6 nM against PfXynC of *P. funiculosum* (Table 1).

The biochemical properties of rice xylanase inhibitors have been elucidated more recently. The rice xylanase inhibitors RIXI, riceXIP, OsXIP, and OsHI-XIP [20,81,82,83] are classified as XIP type. RIXI shares a high protein sequence identity with OsXIP (48%) and riceXIP (57%) [59]. As observed for wheat XIP inhibitors, RIXI inhibits GH11 xylanases from bacterial and fungal origins, but it is inactive against GH10 xylanases from plants [59]. Moreover, the inhibitory activity of RIXI towards family 11 xylanase is positively correlated with the interaction strength of the RIXI–xylanase complex [55]. The efficacy in inhibiting fungal GH11 xylanases is also conserved in OsXIP, as previously reported [83], although the heterologously expressed inhibitor showed partial inhibitory activity against *T. viride* and *T. longibrachiatum* and was ineffective against *Aspergillus niger* GH11 xylanases. Almost complete inhibition was subsequently observed against GH11 xylanase of *Thermomonospora lanuginosus,* which was also used to determine pH and temperature optimal activity for OsXIP inhibition [79]. Interestingly, OsXIP can also inhibit GH10 xylanases, as demonstrated in a recent work in which heterologously expressed OsXIP inhibited the *Aspergillus fumigatus* xylanase A and the catalytic domain of a xylanase from sheep rumen microbiota with *Ki* of 177.94 and 237.37 nM, respectively (Table 1) [69]. Heterologous expression was performed to study the biochemical properties of riceXIP [57]. Kinetics of the GH11 TfxA_CD214 xylanase highlighted a competitive inhibition mechanism with a *Ki* of 12.2 nM (Table 1).

A novel chitinase-like XIP was isolated from the dicot species *Coffea arabica* (CaclXIP). Although the inhibitor has a (β/α)_8_ topology common to class III chitinases as predicted by amino acid sequence, the purified recombinant CaclXIP showed a residual chitinolytic activity and evident inhibition towards the xylanases from *Acrophialophora nainiana* and *A. niger* [84]. Conversely, a rice chitinase-like protein (OsCLP) with a high degree of similarity with TAXI-type XIs isolated and cloned from rice showed strong chitinase activity, but it lacked inhibition activity against the fungal antagonists *Aureobasidium pullulans* or *T. viride* xylanases [85]. However, the high expression level of OsCLP following infection with *Rhizoctonia solani* suggests a role in plant defense against pathogenic fungi.

## 4. Structural Properties of Xylanase-XI Interaction

XIs may be a valuable tool for plant defense if inhibitor proteins display a broad range of activity against the variety of xylanases secreted by phytopathogens. Otherwise, XIs could be structurally modified to enhance their activity and extend their inhibition range. The possibility of engineering new forms of XIs depends on the knowledge of the detailed structure of the xylanase–XI interaction. Here we aim to update the knowledge about the structural determinants for enzymatic inhibition strength and specificity of TAXI, XIP, and TLXI type XIs. We focused on the structural basis of xylanase necrotizing activity inhibition (see below) by XIs and on the structural basis of rice XIs enzymatic inhibition.

For TAXI type XIs, crystallographic analysis of enzyme–inhibitor interaction has been performed only for TAXI-I and TAXI-II in complex with GH11 family *B. subtilis* xylanase A (BsXynA) or *A. niger* xylanase A (AnExlA) [75,86,87]. The crystallographic analysis explains the different inhibition specificity of TAXI-I and TAXI-II towards the two mentioned xylanases that depend on the identity of only a few amino acidic residues.

**Figure 4:**
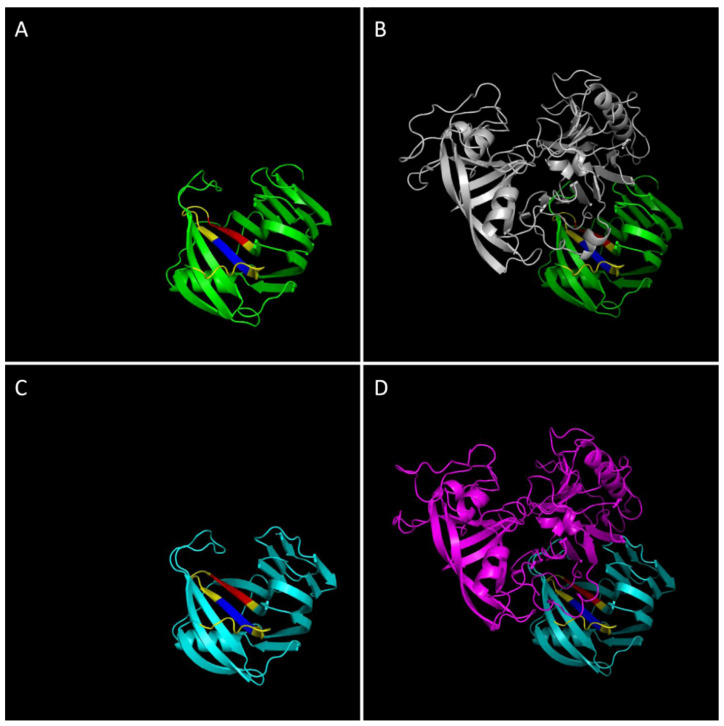
Molecular models of (**A**) *B. cinerea* xylanase BcXyn11a; (**B**) BcXyn11a interacting with TAXI-I; (**C**) *F. graminearum* xylanase FGSG_03634; (**D**) FGSG_03634 interacting with TAXI-III. Hypothetical three-dimensional complexes in B and D are based on the crystal structure (2B42) of TAXI-I in complex with *B. subtilis* xylanase A [75]. Models have been obtained by aligning a single protein structure to 2B42, used as structural templates, by PhMol software (The PyMOL Molecular Graphics System, Version 2.0 Schrödinger, LLC). Green: BcXyn11a. Grey: TAXI-I. Cyan: FGSG_03634. Magenta: TAXI-III. Yellow: 25 aa peptide identified in [90]. Red: M1 peptide (YGWT). Blue: M2 peptide (YYIV).

The molecular basis of the capacity of TAXI-I and TAXI-III to inhibit cell death activity of *F. graminearum* xylanases FGSG_03624, whose ability to induce defense responses in plants has recently been demonstrated [88], and FGSG_10999 [67,89] and *B. cinerea* BcXyn11a [23] is not well understood and therefore requires further studies. In this regard, only one molecular modeling study is available between the inhibitor TAXI-III and the FGSG_03624 xylanase [89]. However, in that model, the short amino acid sequence of FGSG_03624 (139–149, QKKGEINIDGS), identified as the homolog of the hypothetical necrotizing peptide of EIX and BcXyn11A of *T. viride* and *B. cinerea,* respectively [18,19,89], was not interacting with TAXI-III. That finding raised the question of whether the cell death activity of FGSG_03624 was caused by the proposed peptide 139–149 or was located in another protein region. Recently, Frìas et al. (2019) [90] identified a 25-residues necrotizing peptide in the 88–112 region of BcXyn11a that is partially included in the catalytic site of the enzyme and that contains two conserved regions of the four consecutive amino acid residues YGWT and YYIV, named M1 and M2, respectively. *F. graminearum* FGSG_03624 and *B. cinerea* BcXyn11a have a high sequence similarity in the region containing the 25-residues necrotizing peptide [90]. Models of BcXyn11a (Figure 4A), TAXI-I/BcXyn11a complex (Figure 4B), FGSG_03624 (Figure 4C), and TAXI-III/FGSG_03624 complexes (Figure 4D) support the hiding of the 25-residues necrotizing peptide containing both M1 and M2 peptides by TAXI inhibitors. Therefore, TAXI inhibitors can prevent the recognition of the necrotizing peptide by a putative plant receptor and, thus, the activation of necrotic pathways in plant cells.

Currently, a xylanase receptor has been identified only in tomato as an LRR-like protein (glycoprotein with a signal for receptor-mediated endocytosis) able to bind the necrotizing peptide of *T. viride* EIX [91]. Recently, the capacity to inhibit cell death caused by the xylanases FGSG_03624 and FGSG_10999 was also demonstrated for TAXI-II and TAXI-IV [35,39]. The capacity to inhibit xylanase cell death activity is not a prerogative of TAXI-type inhibitors because XIP-I also proved effective in limiting cell death activity of the *F. graminearum* xylanase FGSG_11487 [67]. Interestingly, enzymatic and cell death inhibitory activities are separated features in this xylanase because XIP-I inhibited cell death but not the enzymatic activity of the FGSG_11487 xylanase [67]. The capacity to inhibit the cell death activity of xylanases has implications in plant defense that will be discussed later in the review.

The crystal structures of XIP-I in complex with GH10 *Aspergillus nidulans* AnidXlnC xylanase or, on the other hand, with GH11 *P. funiculosum* PfXynC xylanase, provide the molecular basis for the inhibition by XIP-I. For both GHs, substrate–mimetic contacts and interactions covering the active site demonstrate the competitive inhibition mechanism [72].

These structural studies highlight that XIP-type XIs have two different sites of interaction with GH10 and GH11 xylanases located on the opposite sites of the inhibitor molecule [72]. GH10 bacterial xylanases and fungal *Aspergillus aculeatus* AaXyl2 have two amino acid insertions in the primary structure with respect to AnidXlnC xylanase, which explains their resistance to XIP-type inhibitors. Confirming this hypothesis, all fungal xylanases resistant to the XIP inhibition have two similar insertions [92]. Interestingly, XIP-I shows structural homology with some type III chitinases belonging to the GH18 family classified into the PR-8 class, although XIP-I has no chitinase activity.

Recently, the rice RIXI inhibitor was expressed in *Pichia pastoris* to characterize the molecular basis for the inhibition of GH11 xylanases [55]. Molecular docking and molecular dynamics simulations of the RIXI–xylanase complex showed that the essential region for inhibition of RIXI (amino acid residues 144–153) is the long loop that inserts into the groove between the thumb and the palm region of the GH11 xylanases, as observed for XIP-I–*P. funiculosum* PfXynC complex [72]. The study concluded that the inhibitory mechanism of RIXI is analogous to wheat XIP-I [55]. Weak interaction analysis between RIXI and the recombinant *Bacillus amyloliquefaciens* xylanase A (reBaxA), the recombinant *Thermomonospora fusca* TF xylanase A catalytic domain (TfxA_CD), and their corresponding mutants highlighted that H bonds and van der Waals forces play an essential role in the interaction between the inhibitor and the four xylanases [55]. H bonds and van der Waals forces play a role also in the interaction between OsXIP and the catalytic domain of GH10 XYN-LXY_CD derived from Hu sheep rumen microbiota [69]. OsXIP competitively inhibited XYN-LXY_CD by occupying the catalytic pocket of the xylanase [69]. A similar inhibition mechanism was also observed against the GH10 xylanase A of *A. fumigatus* [54]. Additionally, the mechanism of inhibition of GH11 xylanases by OsXIP is partially elucidated: the long loop (Lα_4_β_5_) of OsXIP occupies the catalytic grooves of *B. amyloliquefacens* BaxA xylanase, preventing access to the substrate [62].

A xylanase-α-amylase inhibitor protein (XAIP) belonging to a new sub-family (GH18C) and showing a sequence identity of 36% to XIP-I was isolated from the underground bulbs of *Scadoxus multiflorus*. This inhibitor showed the ability to inhibit GH11 xylanases and GH13 α-amylases. The crystal structure of XAIP and XAIP-II (another XAIP belonging to the same family) was determined to study the structural basis of the inhibition of xylanases and α-amylases, highlighting that XAIPs inhibit the two types of enzymes through two independent binding sites located on opposite regions of the protein [73,93]. A comparison of the structure of XAIP with that of XIP-I suggests that loops α3–β4 and α4–β5 of XAIP are involved in the binding of GH11 xylanases and that helix α7 and loop β6– α6 are responsible for the interaction with α-amylases. The site of interaction of XIP-I with GH11 xylanases is the loop α3–β5, which occupies the binding cleft of GH11 xylanases. The corresponding loop in XAIP is shorter due to three deletions, making it unsuitable to be inserted in the binding cleft of GH11 xylanase [93]. Further studies provided the molecular basis of the decreased efficacy of XAIP-II against GH11 xylanases compared to XAIP. The loop α3-β3, which is suitable to bind GH11 xylanases in XAIP-II, is stereochemically less favorable for binding GH11 xylanases because of the addition of an extra residue (Ala105) and due to the replacement of two essential residues (His106 and Asn109) of XAIP with Thr107 and Ser110 [73].

Noteworthy, XIP-I has cross-inhibitory activity against two GH13 barley α-amylases [94], suggesting a divergent evolution of XIPs and XAIPs which led to functional diversification.

TLXIs and thaumatins have highly similar tertiary structures. However, the main difference is the number of domains: TLXI contains only two domains, whereas thaumatins have three domains [95]. The key feature of TLXI is domain I, a β-sandwich built up of two β-sheets comprising six (B1 to B6) and five (A1 to A5) β-strands. The occurrence of five disulfide bridges and three β-sheets provides higher stability to the protein than the other two classes of XIs from wheat [80]. However, the crystal structure of a TLXI-xylanase complex is still unavailable.

In contrast to TAXI- and XIP-type XIs, for which competitive inhibition kinetics have been demonstrated, TLXI-type XIs inhibit xylanases in a non-competitive manner by binding outside the active site [31]. The key residue for inhibition activity is His22, located in the flexible loop [96]. In contrast to other PR-5 members with antifungal activity, TLXI does not have a negatively charged surface cleft rich in hydrophilic residues located in thaumatins at the boundary between domains I and II because TLXIs lack domain II. Moreover, although the acidity of the cleft in other PR5 family members is mainly caused by residues located in domain I, TLXI does not contain an acidic region corresponding to the acidic cleft of other PR5 family members [95].

## 5. XI Regulation and Localization

The regulation and expression pattern of wheat XIs is well documented in previous studies, and few novel insights have been provided in the last years. Several cis-acting elements responsive to biotic and abiotic stresses have been previously detected in the promoter regions of TAXI, XIP, and TLXI [33], indicating a conserved stress response function among XIs. A recent study analyzed the promoter region of *TAXI* genes from bread wheat and identified several sequences of cis-acting elements responsive to wounding, pathogen infection, plant hormones (MeJA, ABA, SA, GA, IAA, and ET), and abiotic stresses (drought, high temperature, and low temperature) [39].

Recently, there has been considerable advancement in knowledge on the regulation and expression pattern of rice XIs. Key sequences associated with stress responses and plant hormones have been identified in the promoter of rice *XIs*. The promoter regions’ analysis also provided important information about expression specificity. For example, an investigation of the promoter sequence of *RIXI* highlighted the occurrence of 13 W-boxes, binding sites for WRKY transcription factors [97]. Two WRKY transcription factors, OsWRKY6 and OsWRKY46, were demonstrated to bind *RIXI* promoter, differently affecting RIXI expression. The promoter deletion analysis of another rice XI, *OsXIP*, revealed a 562 bp region (−1451 to −889) as the key sequence for the herbivore’s stress response [98]. An APETALA2/ETHYLENE RESPONSE FACTOR (AP2/ERF) transcription factor OsERF71 and a basic helix-loop-helix protein (OsbHLH59) directly binding to the above-mentioned promoter region were identified as transcriptional regulators of *OsXIP* [98]. Cis-acting elements, such as TGACG motif, ARR1AT, and WRKY71OS, which are responsive to growth regulators, W-box (TGACY) responsive to salicylic acid or wound, G-box (CACGTG) and G-box-like (CANNTG,) which are binding sites of basic helix-loop-helix (bHLH) transcription factors, were found in the promoter region of *OsXIP* [98]. Several cis-acting elements were also found in the promoter region of *riceXIP*, including Myb-binding sites (involved in the transcriptional activation of *PR* genes), W-box sequences, GCC-box related to MeJA responsiveness, and TGACG-motif sequences. Additionally, the analysis of the 5′-deleted mutants of the *riceXIP* promoter suggested the occurrence of cis-elements responsive to MeJA and wounding treatments [58]. This promoter deletion analysis also highlighted the occurrence of two repressor elements (between −1502 and −1147 bp and between −182 and −80 bp, respectively) and an enhancer element (between −1147 and −859 bp) [58].

Unlike TAXI-III, whose localization has been demonstrated in the apoplastic space [21], OsHI-XIP was localized in the endoplasmic reticulum (ER) [20]. The upstream region of *OsXIP* fused with the *GUS* reporter gene highlighted the tissue-specific expression pattern of OsXIP. The inhibitor was highly expressed in the roots and in the veins, but it was weakly expressed in the leaves. GUS activity was found in healthy roots and leaves of young seedlings [79]. The expression in the young seedling and roots is shared with riceXIP, RIXI, TAXI, XIP, and TLXI [33,83], suggesting their role as a defense barrier in organs susceptible to pathogen infection [99].

Tissue-specific along with growth stage-specific expression pattern was also demonstrated for RIXI. A strong expression was found in the vascular bundles, leaves, roots, stems, and leaf cells of young seedlings; the expression was weak in the stamen, at the base of the ovary, and lamina veins; no expression was found in the pistil [22]. Wheat TAXI, XIP, and TLXI are predominantly expressed in wheat grains. However, a weak xylanase inhibitory activity has also been detected in other plant organs such as roots, shoots, and leaves [51]. Some *TAXI* gene families exhibit tissue-specific expression in the early grains, in stems, or roots at different growth stages [39].

## 6. Role of XIs in Plant Defense and Crop Engineering for Plant Protection

Several features indicate that XIs likely contribute to host defense: they share sequence and structure homology with some PR proteins, and their expression is induced during stress conditions. For example, some members of *TAXI* and *XIP* gene families are significantly induced by wounding, herbivore infestation, treatment with SA, JA, MeJA, and deoxynivalenol, and following infection with *Erysiphe graminis, F. culmorum,* and *F. graminearum* [33,39].

Rice XIs are also induced by pathogen infection. For example, Hou et al. (2014) [59] reported a higher RIXI expression after infection of rice seedlings with *P. oryzae*. Wounding or MeJa stresses induce riceXIP expression in both root and shoot tissues. Mechanical wounding and JA, along with herbivore infestation, also induce the expression of OsHI-XIP and Os-XIP [20,79,98]. Conversely, RIXI expression is not observed following MeJA or wounding treatment [83], indicating a possible functional diversification among rice XIs.

Above all, the involvement of XIs in plant defense is supported by their demonstrated capacity to increase plant resistance to pathogens in engineered crops. An example is represented by the overexpression of TAXI-III in durum wheat transgenic plants, which reduced disease symptoms caused by *F. graminearum* [21]. The resistance provided by TAXI-III was associated with the inhibitor’s ability to limit enzymatic and cell death activities of *F. graminearum* xylanases FGSG_03624 and FGSG_10999 [67,89,100]. The capacity of XIs to inhibit cell death activity of xylanases secreted by pathogens proved crucial in transgenic *Arabidopsis* plants expressing TAXI-I. These plants showed increased resistance to *B. cinerea* by inhibiting the necrotizing activity of BcXyn11a, although the provided protection was dose-dependent [23]. Due to the dichotomy between enzymatic and cell death activities of xylanases, these results raise the question of which of these features is essential for pathogenesis. Considering this aspect, the concomitant inhibition by XIs of cell death and of enzymatic degradation of xylan seems important to increase plant resistance to pathogens. However, no studies investigated whether the inhibition of the enzymatic or necrotizing activity is critical for increasing plant resistance.

An additive effect of resistance was obtained by stacking TAXI-III and PvPGIP2 (a PGIP from *Phaseolus vulgaris*) in durum wheat, resulting in enhanced disease resistance against *F. graminearum* compared to parental lines individually carrying TAXI-III or PvPGIP2 [24]. These results indicate the feasibility of increasing plant resistance by pyramiding inhibitors with a protective role on different cell wall components, namely xylan and pectin.

**Figure 5:**
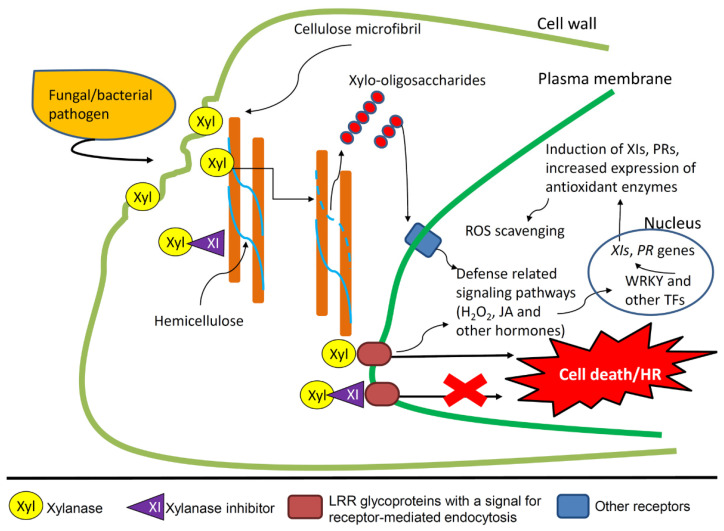
Schematic representation of the inhibitory activity of XIs towards bacterial or fungal xylanases and defense responses activated by the xylanase-xylanase inhibitor interaction. The enzymatic inhibition by XIs prevents hemicellulose degradation and cell wall disassembly. The release of xilo-oligosaccharides (XOS) by the activity of xylanases likely induces plant defense responses through their recognition by plant receptors. Additionally, XIs prevent necrosis of plant cells likely triggered by the binding of xylanases with a putative receptor. Currently, a receptor has been identified only in tomato as an LRR-like glycoprotein [91]. The interaction of XIs with xylanases amplifies defense-related pathways, such as H_2_O_2_ release and JA-mediated signaling, which lead to increased expression of XIs, PRs, and antioxidant enzymes, as demonstrated in rice overexpressing RIXI [22]. The antioxidant enzymes catalase and superoxide dismutase have a role as ROS scavenging to decrease oxidative stress.

The efficacy of XIs to counteract pathogen infections has also been demonstrated in rice overexpressing Os-XIP or RIXI, which showed enhanced disease resistance to *P. oryzae* and increased *PR* gene expression via JA-mediated signaling pathways [22,79]. Moreover, RIXI expression was accompanied by higher levels of hydrogen peroxide and more considerable changes in catalase and superoxide dismutase activities, scavenging the excess of reactive oxygen species [22]. An overview of the immune responses associated with XIs is reported in Figure 5. In the induction of plant immunity, an essential role is also played by the release of xylooligosaccharides (XOS) acting as DAMPS as the result of arabinoxylan degradation by xylanases [101]. For XIs, it could be hypothesized a mechanism similar to that occurring with PGIP inhibitors, which leave a residual activity to polygalacturonases determining the slow release of oligalacturonides sensed by the plant as DAMPS [46,102].

Rice XIs also play an essential role in resistance against herbivores. Indeed, rice lines overexpressing OsHI-XIP showed a reduced larval infestation of the rice striped stem borer *Chilo suppressalis* and decreased feeding and oviposition preferences of the rice brown planthopper *Nilaparvata lugens* [20]. Interesting results were obtained with CaclXIP from *Coffea arabica* that, when expressed in transgenic soybean plants, showed the ability to inhibit spore germination of the fungal pathogen *Phakopsora pachyrhizi*, the causal agent of soybean Asian rust [84]. Another candidate to be exploited for plant protection could be the xylanase inhibitor-like protein (XILP) from sorghum, inhibiting the mycelial growth of several phytopathogenic fungi, including *Fusarium oxysporum*, *Alternaria solani*, *Verticillium dahliae,* and *Bipolaris maydis* [103].

Overall, the results achieved with the expression of XIs by transgenic approach are very promising and open new scenarios in the use of these inhibitors in crop protection. Indeed, XIs could be expressed in combination with other defense components by gene stacking to integrate different resistance mechanisms and reduce the risk of loss of efficacy. Noteworthy, the transgenic expression of XIs in plant tissues that naturally do not express XIs can determine silencing events [21]. This mechanism likely prevents the alteration of plant growth and development processes and maintains plant homeostasis.

As an alternative to the transgenic approach, the effectiveness of XIs in plant protection could be increased by assisted evolution.

## 7. Strategies to Reduce the Effect of XIs in Food Processing and Quality

Fungi belonging to the genus *Aspergillus,* such as *A. niger* and *A. awamori,* are important sources of xylanases for industrial processes. Members of this genus are also used for large-scale production of cellulases, pectinases, α-amilases, and β-glucosidases [5]. Xylanases are effectively applied in various industrial applications, including feed, food, textile, pulp, and paper. Xylanases have additionally been suggested to provide valuable possibilities for the bioconversion and biodegradation of diverse agro-industrial and processing wastes into numerous value-added products [104]. However, the presence of XIs can have a negative effect on several industrial processes in which adding microbial xylanases improves the quality of the final product [65]. For example, adding microbial xylanases in poultry feeds is a common practice for improving feed digestion. Indeed, non-starch polysaccharides such as xylans increase the viscous intestinal environment reducing the digestion rate of nutrients and contributing to animal diseases. Pelleting poultry feed at high temperatures could be a strategy to decrease XI inhibitory activity (Table 2). However, at high temperatures (>85 °C), XI activity is only partially reduced, and a much more substantial decrease in endogenous xylanase activity is observed [105]. In these conditions, adding microbial xylanases does not affect the release of reducing end sugars, thus indicating that the XI activity is still high [105].

The quantification of wheat-grain XI content is a crucial quality parameter to make informed choices on wheat cultivars and to improve resource-use efficiency in broiler diets supplemented with feed xylanase (Table 2). Krogh Madesen et al. (2018) [106] analyzed 32 wheat cultivars for inhibition of the commercial xylanase Ronozyme WX. The wheat cultivars were also evaluated for the contents of non-starch polysaccharides and amino acids. Four cultivars with different levels of inhibitory activity were selected for a broiler feeding experiment. Broilers fed with grains of a cultivar with high inhibitory activity (Hereford) showed a 7% weight decrease (day 14) compared to broilers fed with grains of a cultivar with less inhibitory activity (Diantha). These results highlight that wheat cultivars should be considered for xylanase inhibition levels to ensure high and consistent growth rates in broilers and in other bred species, such as pigs, for which xylanase is used as feed supplementation. In general, the level of xylanase inhibition should be evaluated in all vegetal matrices used for agro-industrial processes in which xylanases are applied to improve the degradation of hemicellulose. This would allow an improvement in the degradation efficiency of xylanases.

The search for new xylanases that escape inhibition by XIs, with high thermo-stability and specific activity, is the most promising strategy to make the use of xylanases more efficient in biotechnological applications (Table 2). Two GH10 xylanases of the fungus *Chrysosporium lucknowense* tested against water extracts of rye flour demonstrated high resistance to the inhibition of XIs and high activity against arabinoxylan [64]. The tolerance to inhibition was ascribed to the presence of insertions in the loop β_8_–α_8_ that are typical for bacterial but not for fungal xylanases. Of the two xylanases, Xyn10B was characterized by higher specific activity against arabinoxylans than Xyn10C. The lower specific activity of the latter was attributed to the presence of side L-arabinose residues in the backbone of arabinoxylan [64].

The genetic engineering approach is an attractive strategy to increase the variability and functionality of xylanases used for industrial applications (Table 2). Heterologous expression of the GH10 xylanases PcXylA from *Penicillium canescens* and TrXyn3 from *Trichoderma reesei,* with an insertion of 5 amino acid residues into the peptide loop forming the active site cleft, reduced the inhibition of recombinant xylanases by the XIs from rye compared to corresponding wild-type xylanases [92]. On the other hand, the authors detected a moderate decrease in thermostability and in specific activity against glucuronoxylan for PcXylA-ins and TrXyn3-ins because of the insertion. An interesting opportunity is the heterologous expression of cereal xylanases that are not inhibited by XIs. EXY3 and EXY4 xylanases from wheat cultivar “Chinese Spring”, both containing the catalytic domain of GH10 xylanases, were successfully expressed and showed enzymatic activity against beechwood xylan, highlighting a potential application in cereal-based processes [107].

XOSs are obtained from treatments of lignocellulosic materials or isolated xylan with endo-xylanases. These oligomers are used as ingredients in functional foods [108] because of their various physiological properties, such as the production of short-chain fatty acids [109] and the growth promotion of *Bifidobacterium*, a probiotic bacterium that can decrease blood pressure and blood sugar levels [110,111]. The search and the bioengineering of new endo-xylanases with improved enzymatic efficiency are fundamental for their use in the commercial production of XOS. In this regard, Xu et al. (2016) [112] obtained a mutant (reBaxA50) of the recombinant *B. amyloliquefaciens* xylanase A (reBaxA). reBaxA50 showed 3.5-fold higher specific activity and improved thermostability as compared to recombinant reBaxA. Moreover, the reBaxA50 hydrolyzed birchwood and oat spelt xylans into XOS at a lower polymerization degree than reBaxA and was more stable than reBaxA under extreme pH and thermal conditions. The higher efficiency of this xylanase was attributed to the mutation of the amino acid residue 138 (S138T). 

**Table 2:** Strategies to reduce the negative effects of XIs in agro-industrial processes.

Strategy	References
Heat treatment of matrices containing XIs	[105]
Selection of plant row materials with low xylanase inhibitory activity	[106]
Application of xylanases that escape the inhibition of xylanase inhibitors	[64]
Engineering xylanases that escape the inhibition of xylanase inhibitors and/or with improved catalytic activity and thermostability	[60,92,107,112,113,114]
Adjustment of xylanase dosage	[115,116]
Targeted disruption of specific XIs in plant species used in agro-industrial processes	[42]

Noteworthy, reBaxA50 exhibited higher residual activity (55.6%) as compared to the corresponding control reBaxA (30.3%) in inhibition assays with the rice recombinant RIXI, providing a further benefit for its use in industrial applications [55]. Similarly, the substitution of the N-terminus of the *A. niger* xylanase A (AnxA) with the corresponding region of *T. fusca* xylanase A (TfxA) determined lower inhibition activity by RIXI and higher thermostability and catalytic activity as compared to AnxA [60,113]. Only five amino acids located in one of the regions of the N-terminus were demonstrated to determine the higher efficiency of the xylanase [114].

Microbial xylanases are also frequently used in cereal-based applications, especially in bread making, for their positive effect on dough properties and bread volume [115]. This effect is associated with the partial conversion of water-unextractable arabinoxylan (WU-AX) to water-extractable arabinoxylan (WE-AX) and to solubilization of WU-AX during processing [36]. The release of WE-AX and solubilization of WU-AX increase the viscosity of the dough with a beneficial effect on the quality of the final product. However, xylanases also degrade WE-AX and solubilize AX (S-AX), which determines a decrease in viscosity.

The xylanase dosage is a key factor for the use of xylanases in bread making, from both economic and sustainability points of view (Table 2). Optimal enzyme dosage is necessary for the maximal increase in bread loaf volume, and it greatly impacts the product’s end quality. The choice of the optimal dosage depends on the activity profiles of xylanases during the bread-making process. Recently, the activity profile of the *B. subtilis* xylanase XBS and its mutants differing in inhibitory sensitivity against XIs during bread making were evaluated for their impact on the xylanase activity, solubilization of WU-AX, and specific loaf volume [116]. Xylanases with higher inhibitory sensitivity required an increase in enzyme dosage compared to xylanase with lower inhibitory sensitivity. Besides, they were only active during the mixing phase and at the beginning of fermentation. Conversely, the less inhibited xylanase hydrolyzed arabinoxylan until the end of fermentation. Increased sensitivity to inhibition by XIs reduces the time interval in which enzymatic activity is functional and, therefore, significantly increases the dosage needed to determine a similar loaf volume. The study also showed that adding xylanases affected starch−starch and starch−gluten interactions due to a decreased water-holding capacity of arabinoxylan, with a positive effect on bread loaf volume.

A promising strategy to reduce the negative effect of XIs on dough quality could be the targeted disruption of specific XIs in elite wheat cultivars (Table 2). As an example, the disruption of the three homologous genes, *XIP-6A*, *XIP-6B*, and *XIP-6D,* in the variety “Fielder” by CRISPR/Cas9 significantly increased dough stability time and SDS-sedimentation values of common wheat [42].

Finally, the use of XIs could positively impact the brewing industry because of the capacity to mitigate premature yeast flocculation during the fermentation of microbially contaminated barley malt [117].

## 8. Involvement of XIs in Food Allergy

An important but little-explored aspect of XIs is their possible role in food allergy. As previously mentioned, XIs are widely present in cereals, especially in wheat. Cereals are a staple food of the human diet, but their consumption can trigger IgE-mediated food allergies. The incidence of food allergies is increasing worldwide, and its prevalence is thought to be 10% of the US population [118]. Among wheat allergens, XIs could be triggering factors. Indeed XIP-I contained in water/salt-soluble extracts from wheat flour was identified as a potential wheat flour allergen in immunoblotting assays with pooled sera from patients with atopic dermatitis who possessed IgE specific to wheat [119]. Another study evaluated the allergenic potential of rice seed proteins identified by MS-based proteomics and analyzed by IgE-binding assay [120]. Among the nine detected proteins, a rice sequence matching with a wheat chitinase-like xylanase inhibitor was positive in the IgE-binding assay [120]. However, more studies are needed to evaluate the impact of XIs on food allergies. In this regard, XIs could be expressed in heterologous systems or purified from plant tissues and tested in a degranulation assay against human sera of patients with food allergies [121].

## 9. Conclusions

The accumulated knowledge of the last years highlights the great genetic variability of XIs, represented by a wide distribution of *XI* genes and allelic variants in all major cereal crops. Unfortunately, this reservoir is largely under-exploited in breeding programs for disease resistance. Increasing the knowledge of the mechanisms underlying disease resistance would allow fine-tuning of their use in plant protection without compromising plant growth and development.

The efficacy of XIs in reducing disease symptoms is documented against biotrophic, necrotrophic, and hemibiotrophic fungal pathogens and herbivores but not against bacteria and oomycetes. In this regard, it would be helpful to verify the inhibition specificity of XIs against xylanases secreted by bacteria and oomycetes before their in vivo expression in plant tissues.

As we showed in this review, much has been done to characterize the inhibition specificity of the identified XIs. However, searching in available germplasms for novel XIs with broader and stronger inhibition activities would be advisable. Another option is the artificial evolution of XIs through a genome editing approach to generate inhibitors with modified inhibition specificities. To date, this strategy is still poorly explored.

Overall, using XIs as a tool for crop protection should consider the possible impact on the subsequent industrial processing of raw materials. The strategies to reduce the negative impact of XIs are essentially based on the search for novel xylanases or on the construction of recombinant xylanases escaping inhibition by XIs. A further poorly explored solution could be the tissue-specific expression of XIs only in plant organs susceptible to pathogen infection. Limiting the accumulation of XIs in tissues and organs used in agro-industrial processes would determine a reduction in the added amount of xylanase, an increased degradation efficiency, and an economic return.

As we reported in this review, some studies indicate XIs as potential allergens. However, the evidence is still poorly investigated, and further studies are needed to evaluate the allergenic potential of XIs.

Finally, some works investigated the timing of the accumulation of XIs in monocot plants, but little is known about their involvement in growth and development. Future research should deepen this aspect.

## Figures and Tables

**Table 1:** Inhibitory activity and *Ki* values of wheat (TAXI, XIP, TLXI), rice (RIXI, riceXIP, OsXIP, OsHI-XIP), and *Scadoxus multiflorus* (XAIP) xylanase inhibitors against xylanases.

Xylanase	GH	Origin	TAXI-I	TAXI-II	TAXI-III	TAXI-IV	XIP-I	TLXI	RIXI	riceXIP	OsXIP	OsHI-XIP	XAIP	References
*Aspergillus aculeatus* (AaXyl2)	10	F	no	no			no	no						[31,44,49,53]
*Aspergillus fumigatus* xylanase A (AfXylA10)	10	F									177.94			[54]
*Aspergillus nidulans* (AnidXlnC)	10	F	no	no			9							[44,49,53]
*Aspergillus niger* (XynIII)	11	F					yes							[41]
*Aspergillus niger* (AnExlA/AnxA/ANX)	11	F	20.1	no			317	135	yes	yes				[31,44,49,53,55,56,57,58,59,60,61]
*Aspergillus niger* hybrid mutant of AnxA xylanase and TfxA_CD (FSI-124)	11	F/B							yes					[60]
*Aspergillus niger* (AnM4Xyl)	11	F			18.8									[21]
*Aspergillus niger* (AnXynA)	10	F	no	no			yes	no	yes					[31,49,53,59]
*Aspergillus niger* (Xyn10)	10	F					yes							[41]
*Aspergillus niger* xylanase hybrid of AnxA xylanase and TfxA_CD (Atx)	11	F/B							yes					[55,60]
*Aspergillus oryzae* (AoXyn)	10	F	no	no			17	no						[31,44,53]
*Bacillus agaradhaerens* (BaXyl)	11	B					no							[44]
*Bacillus amyloliquefaciens* (reBaxA)	11	B							yes	yes	54.09			[55,57,60,62]
*Bacillus amyloliquefaciens* xylanase mutant of reBaxA (reBaxA454)	11	B								yes				[57]
*Bacillus amyloliquefaciens* xylanase mutant of reBaxA (reBaxA50)	11	B							yes					[55,60]
*Bacillus amyloliquefaciens* xylanase mutant T331 of reBaxA (DS199)	11	B									12.16			[62]
*Bacillus* sp.	10	B					no							[44]
*Bacillus subtilis* (BsXynA)	11	B	16.7	yes			no	no						[31,44,49,53,56]
*Bacillus subtilis* (BSX)	11	B	yes	yes					yes					[59,61]
*Botrytis cinerea* (BcXyn11a)	11	F	6	no			2.1							[63]
*Chrysosporium lucknowense* (ClXynB)	10	F	no	no			no							[64]
*Chrysosporium lucknowense* (ClXynC)	10	F	no	no			no							[64]
*Fibrobacter succinogenes* (FsXynC)	11	B					no							[44,53]
*Flavobacterium* sp. MSY-2 (rXFH)	10	B	no				no							[65]
*Fusarium graminearum* (FCSG_11487)	10	F	no	no	no	no	no	no						[39,66,67]
*Fusarium graminearum* (FGSG_03624)	11	F	yes	yes	2.59	yes	no	no						[21,39,66,67,68]
*Fusarium graminearum* (FGSG_10999)	11	F	yes	yes	12.04	yes	no	no						[39,66,67,68]
*Fusarium graminearum* (FGSG_11304)	10	F	no	no	no	no	83.7	no						[39,66,67]
Hu sheep rumen microbiota (XYN-LXY_CD, catalytic domain)	10	B									237.37			[69]
*Hypothenemus hampei* (HhXyl)	10	I					yes							[70]
*Neocallimastix patriciarum* (NpXynA)	11	F	no				no							[71,72]
*Neocallimastix* sp. GMLF1 (Xyn1B)	11	F					no							[71]
*Oryza sativa* (OSX)	10	P							no					[59]
*Paenibacillus sp*	U	B					yes							[41]
*Penicillium funiculosum* (not specified)	11	F											yes	[73]
*Penicillium funiculosum* (PfXynA)	7	F	46	46			106							[74]
*Penicillium funiculosum* (PfXynB)	11	F	2.9	no			89.7							[75,76]
*Penicillium funiculosum* (PfXynC)	11	F	16	17			3.4	289.6						[31,44,49,75,77,78]
*Penicillium funiculosum* (PfXynD)	10	F	no	no			yes							[74]
*Penicillium griseofulvum* (PgXynA)	11	F					no							[77]
*Penicillium occitanis* Pol6 (PoXyn3)	11	F	yes											[78]
*Penicillium purpurogenum* (PpXynA)	10	F	no	no				no						[31,49,53]
*Penicillium purpurogenum* (PpXynB)	11	F	yes	yes										[49,53,75]
*Pseudoalteromonas haloplanktis* TAH3A (XPH)	8	B	no	no			no	no						[65]
*Pseudomonas fluorescens* (PfXynA)	10	B					no	no						[31,44]
*Talaromyces emersonii* xylanase (TeX-1)	10	F	no	no										[49]
*Thermobacillus xylanilyticus* (TxXyl)	11	B						234.7						[31]
*Thermobacillus xylanilyticus* (TxXynA)	10	B						no						[31]
*Thermomonospora fusca* (TFX)	11	B							no					[59]
*Thermomonospora fusca* TF xylanase A catalytic domain (TfxA_CD)	11	B							yes	yes				[55,57]
*Thermomonospora fusca* catalytic domain TfxA_CD mutant (TfxA_CD214)	11	B							yes	12.2				[55,57,79]
*Thermomonospora fusca* catalytic domain TfxA_CD mutant (TfxA_CD309)	11	B							yes	yes				[55,57]
*Thermomonospora fusca* catalytic domain TfxA_CD mutant (TfxA_CD311)	11	B							yes	yes				[55,57]
*Thermomonospora fusca* catalytic domain TfxA_CD mutant (TfxA_CD467)	11	B								yes				[57]
*Thermomonospora fusca* catalytic domain TfxA_CD mutant (TfxA_CD526)	11	B								yes				[57]
*Thermomonospora lanuginosus* (TLx)	11	F							yes	yes	yes	yes		[20,55,57,60,79]
*Trichoderma harzianum* (ThXyn1)	11	F					yes							[41,71]
*Trichoderma longibrachiatum* (endo-1,4-β; M3)	11	F							yes		yes	yes		[20,60,79]
*Trichoderma longibrachiatum* (Xyn I)	11	F						4.2						[31]
*Trichoderma longibrachiatum* (Xyn II)	11	F						no						[31]
*Trichoderma reesei* (TRx)	11	F							yes	yes				[55,57]
*Trichoderma reesei* (Xyn1)	11	F	yes	yes										[75]
*Trichoderma reesei* (Xyn2)	11	F	yes	yes										[75]
*Trichoderma viride* (TvXyl)	11	F	yes	yes			610	170.4						[31,44,49,53,75]
*Triticum aestivum* xylanase (from flour)	10	P	no				no							[49,51]
Uncultured bacterium (rXyn8)	8	B	no	no			no	no						[65]

Available inhibition constants (*Ki*) are expressed in nM. Absence/presence of inhibition is indicated by no/yes. Empty: not available. U: unknown, B: bacterial, F: fungal, P: plant, I: insect.

## Data Availability

All data are available from the corresponding author upon request.

## Supplementary Materials

The following supporting information can be downloaded at: https://www.mdpi.com/article/10.3390/ijms232314994/s1, Figure S1: Phylogenetic tree of the sorghum TAXI-type (A) and XIP-type (B) protein sequences. TAXI and XIP sequences are in black color font; sorghum chitinase sequence used in the XIP phylogenetic tree is in red color font; Figure S2: Phylogenetic tree of the maize TAXI-type (A) and XIP-type (B) protein sequences. TAXI and XIP sequences are in black color font; maize chitinase sequences used in the XIP phylogenetic tree are in red color font; Figure S3: Phylogenetic tree of the rice TAXI-type (A) and XIP-type (B) protein sequences. TAXI and XIP sequences are in black color font; rice chitinase sequences used in the XIP phylogenetic tree are in red color font; Figure S4: Schematic representation of the genomic organization of TAXI family members in the sorghum genome. SbChr, chromosome; Figure S5: Schematic representation of the genomic organization of TAXI family members in the maize genome. ZmChr, chromosome; Figure S6: Schematic representation of the genomic organization of TAXI family members in the rice genome. OsChr, chromosome; Figure S7: Schematic representation of the genomic organization of XIP family members in the sorghum genome. SbChr, chromosome; Figure S8: Schematic representation of the genomic organization of XIP family members in the maize genome. ZmChr, chromosome; Figure S9: Schematic representation of the genomic organization of XIP family members in the rice genome. OsChr, chromosome; Figure S10: Basic Local Alignment Search Tool (BLAST) search results using (A) TAXI-I (AJ438880.1) and (B) XIP-I (ADB81849.1) as a query on National Center for Biotechnology Information (NCBI).
