# Supplementary material for: Xylanase Inhibitors: Defense Players in Plant Immunity with Implications in Agro-Industrial Processing
Source: Int J Mol Sci. 2022 Nov 30;23(23):14994. doi: 10.3390/ijms232314994 (PMC9739030; doi:10.3390/ijms232314994)
Supplement: Supplementary file 1 [file ijms-23-14994-s001.zip › Supplementary Figures_.pdf]

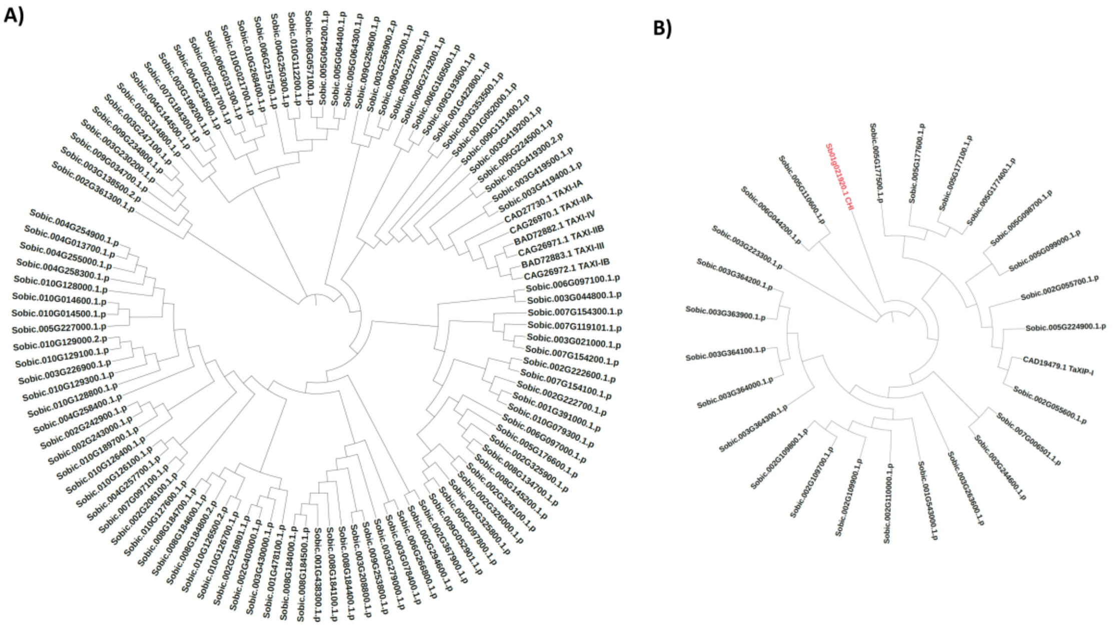

Figure S1: Phylogenetic tree of the sorghum TAXI-type (A) and XIP-type (B) protein sequences. TAXI and XIP sequences are in black color font; sorghum chitinase sequence used in the XIP phylogenetic tree is in red color font.

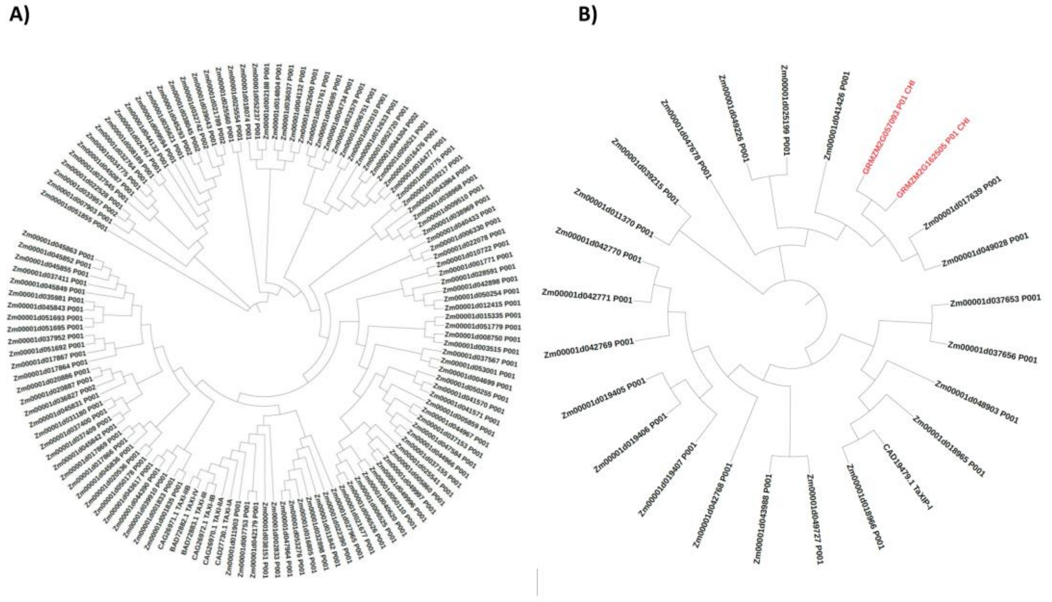

Figure S2: Phylogenetic tree of the maize TAXI-type (A) and XIP-type (B) protein sequences. TAXI and XIP sequences are in black color font; maize chitinase sequences used in the XIP phylogenetic tree are in red color font.

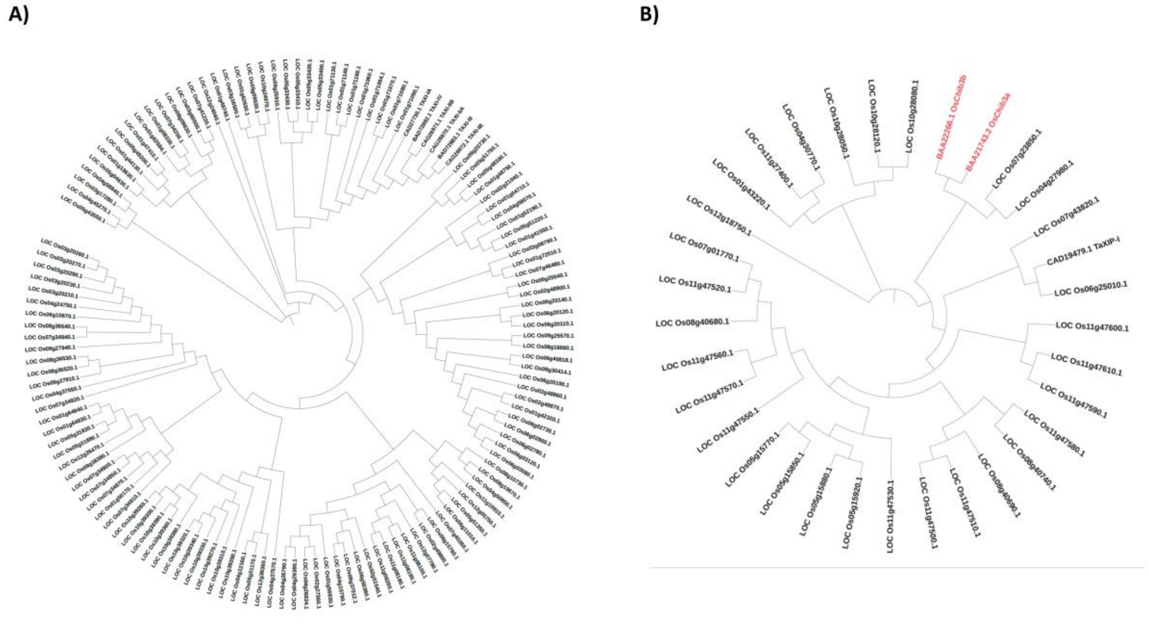

Figure S3: Phylogenetic tree of the rice TAXI-type (A) and XIP-type (B) protein sequences. TAXI and XIP sequences are in black color font; rice chitinase sequences used in the XIP phylogenetic tree are in red color font

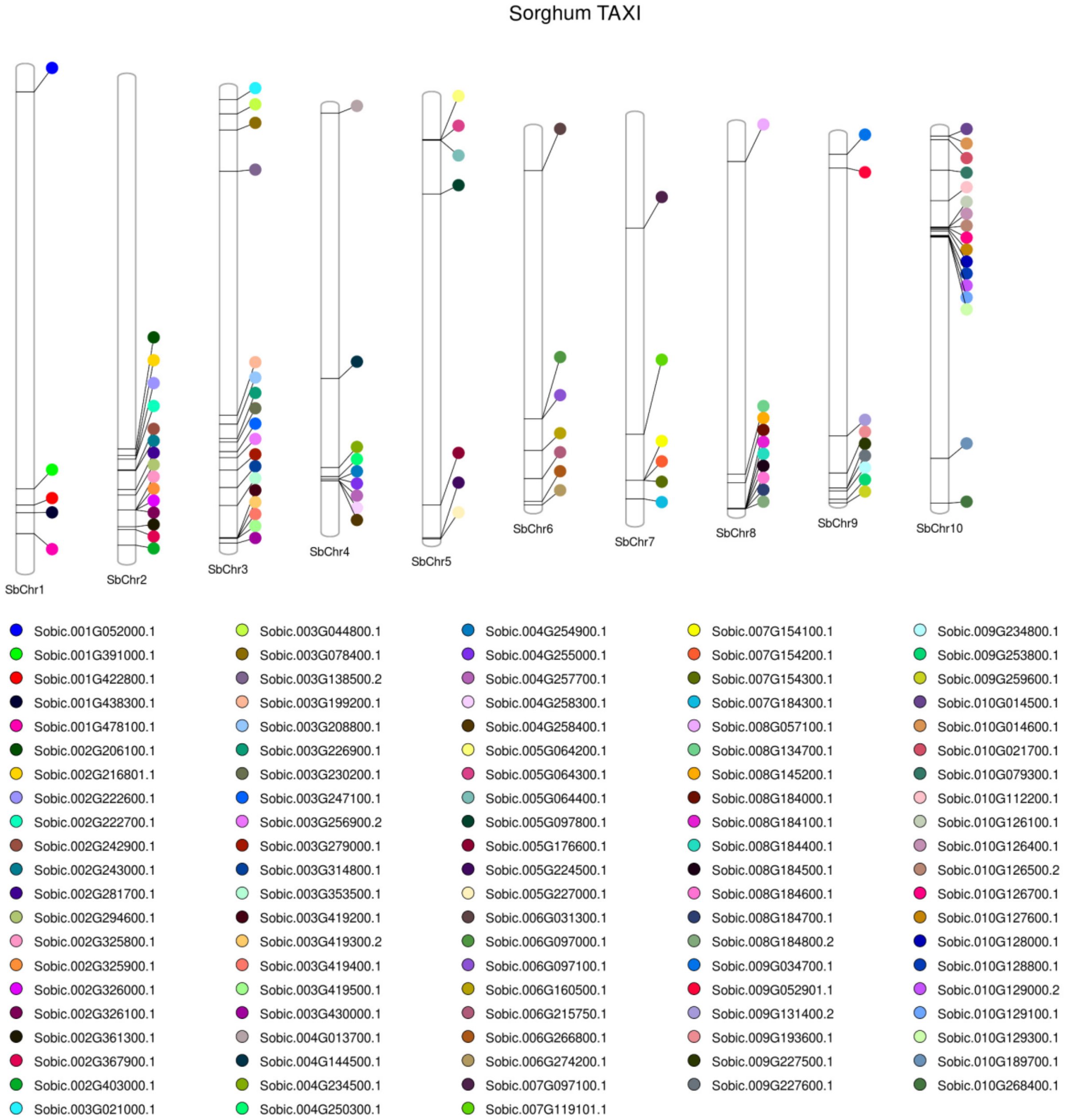

Figure S4: Schematic representation of the genomic organization of TAXI family members in the sorghum genome. SbChr, chromosome.

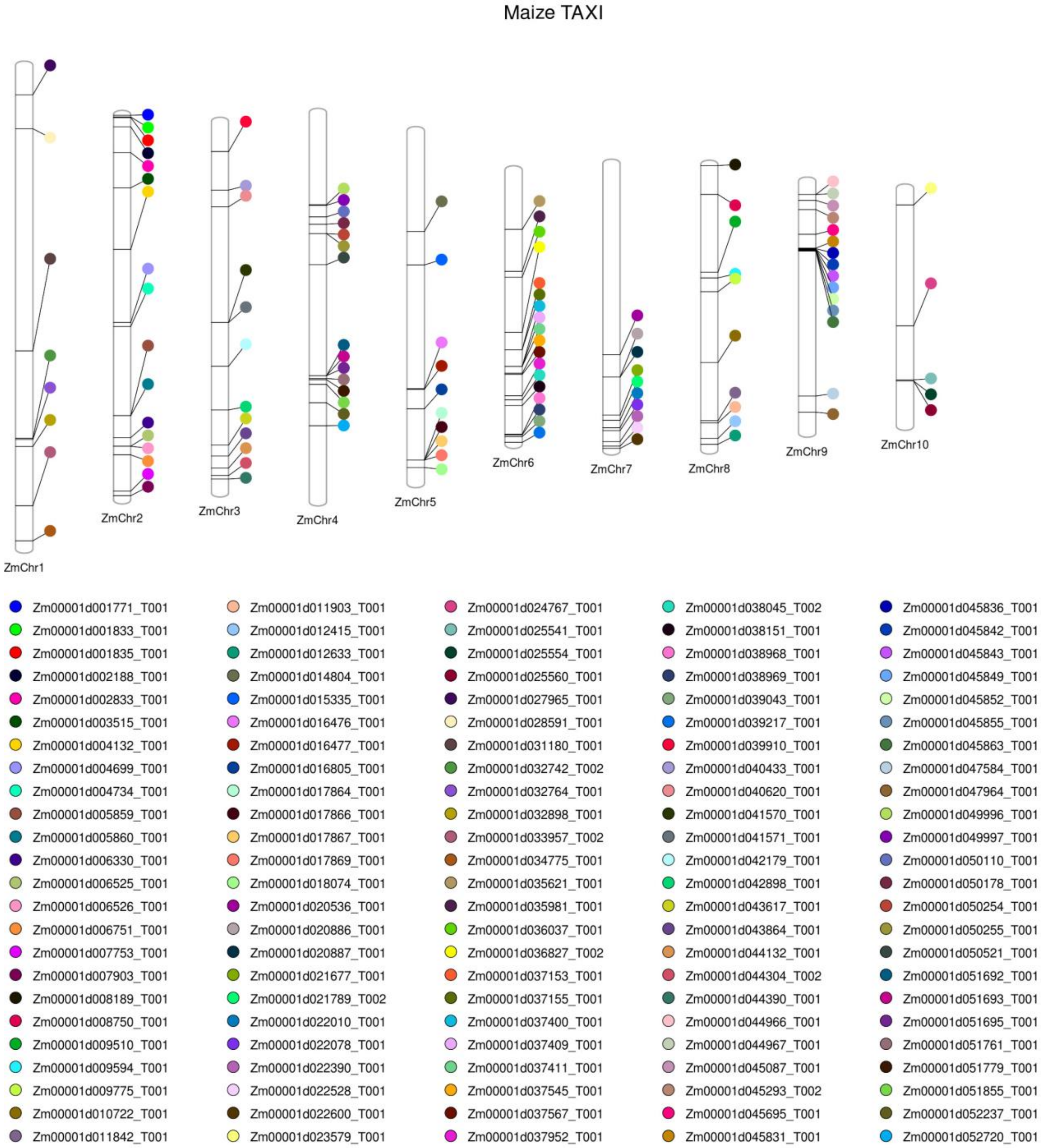

Figure S5: Schematic representation of the genomic organization of TAXI family members in the maize genome. ZmChr, chromosome.

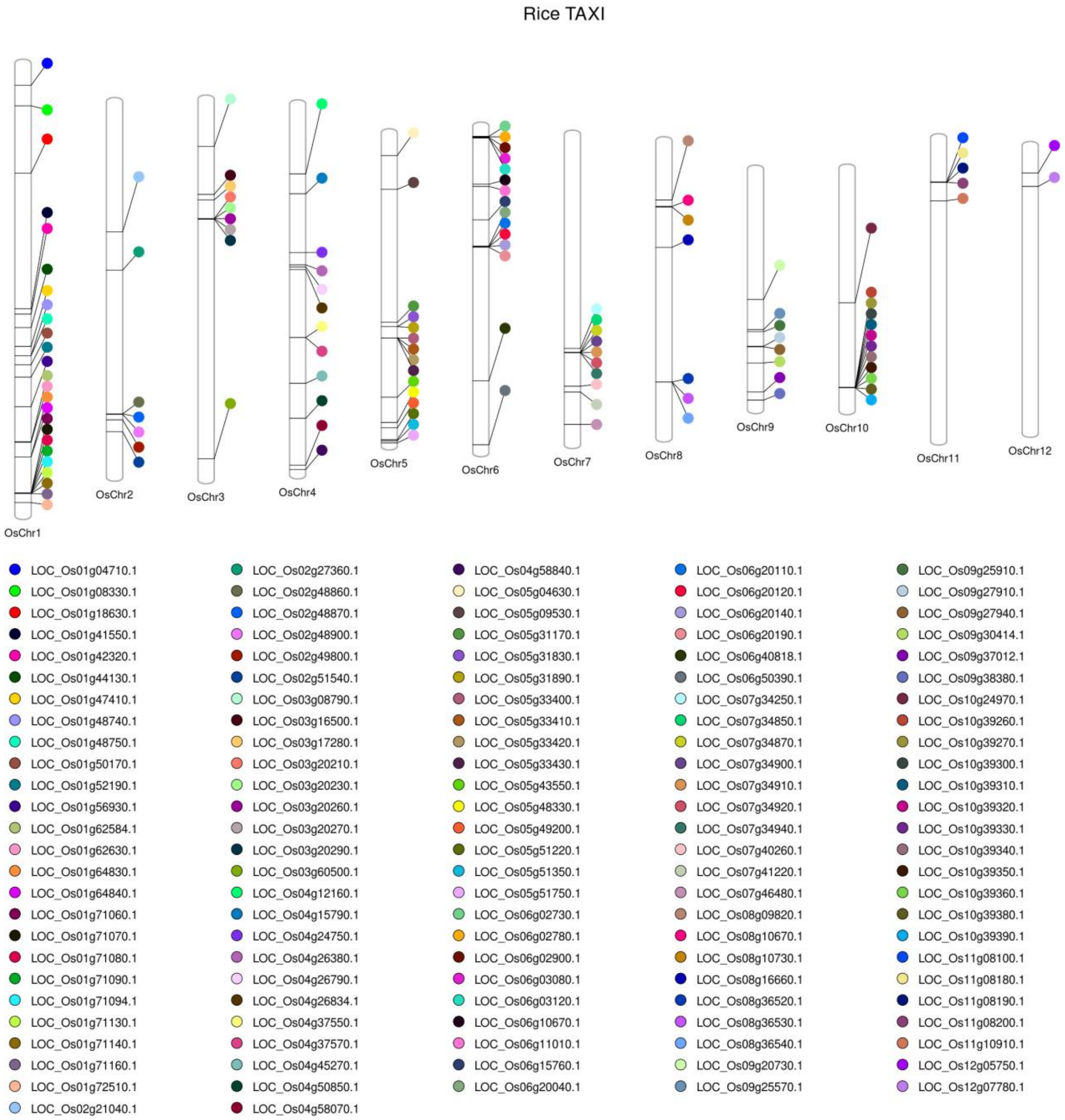

Figure S6: Schematic representation of the genomic organization of TAXI family members in the rice genome. OsChr, chromosome.

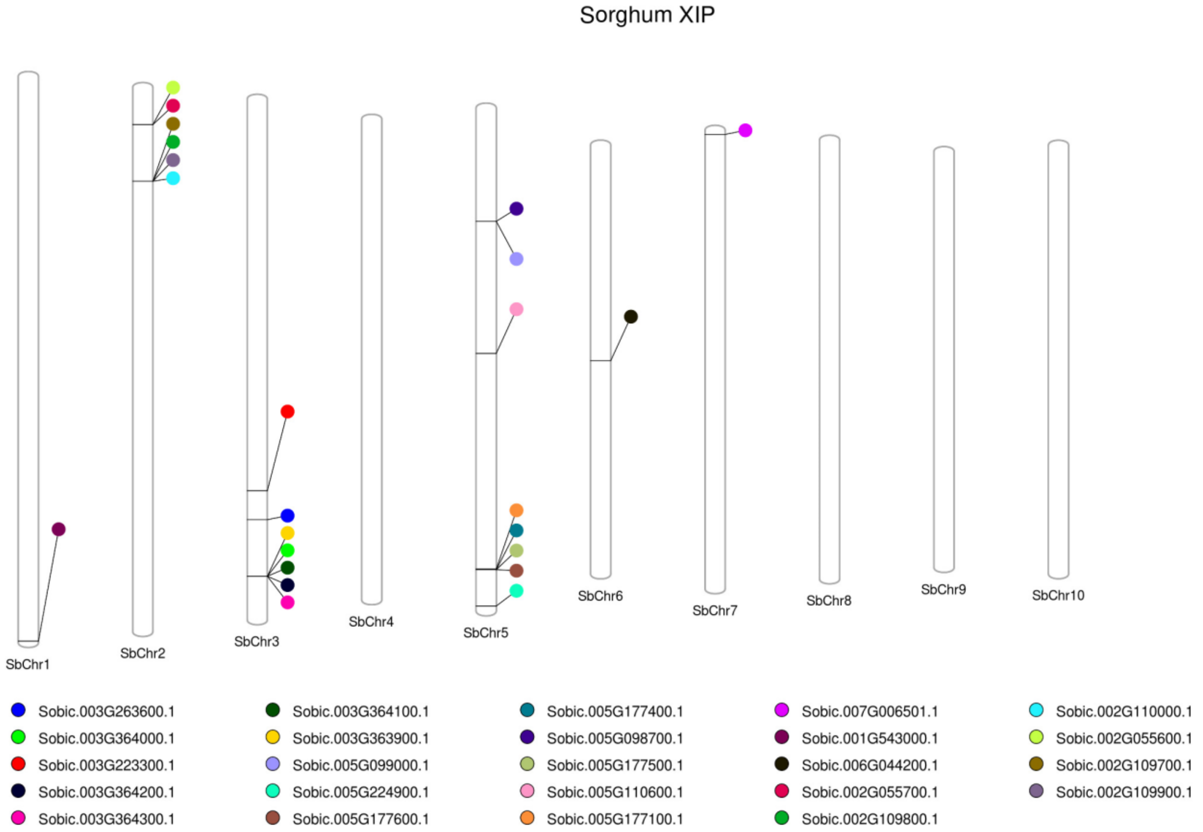

Figure S7: Schematic representation of the genomic organization of XIP family members in the sorghum genome. SbChr, chromosome.

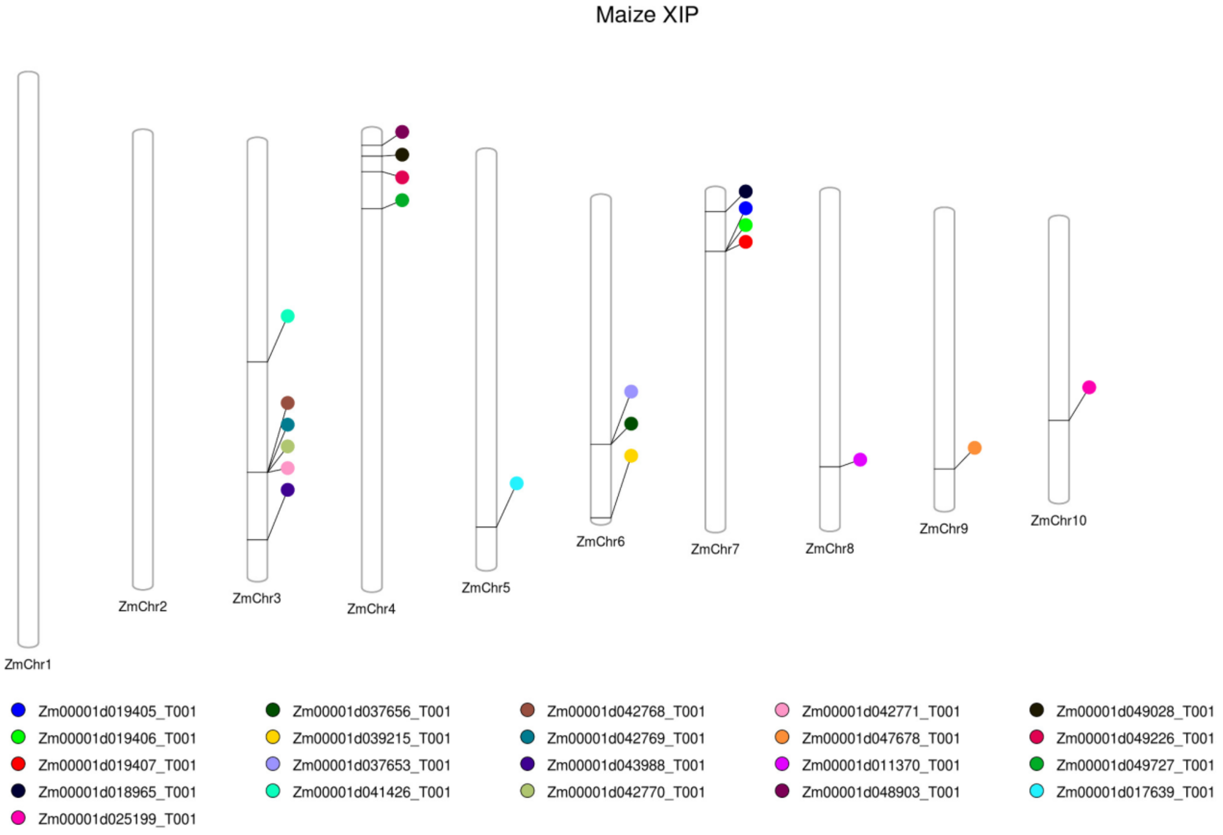

Figure S8: Schematic representation of the genomic organization of XIP family members in the maize genome. ZmChr, chromosome.

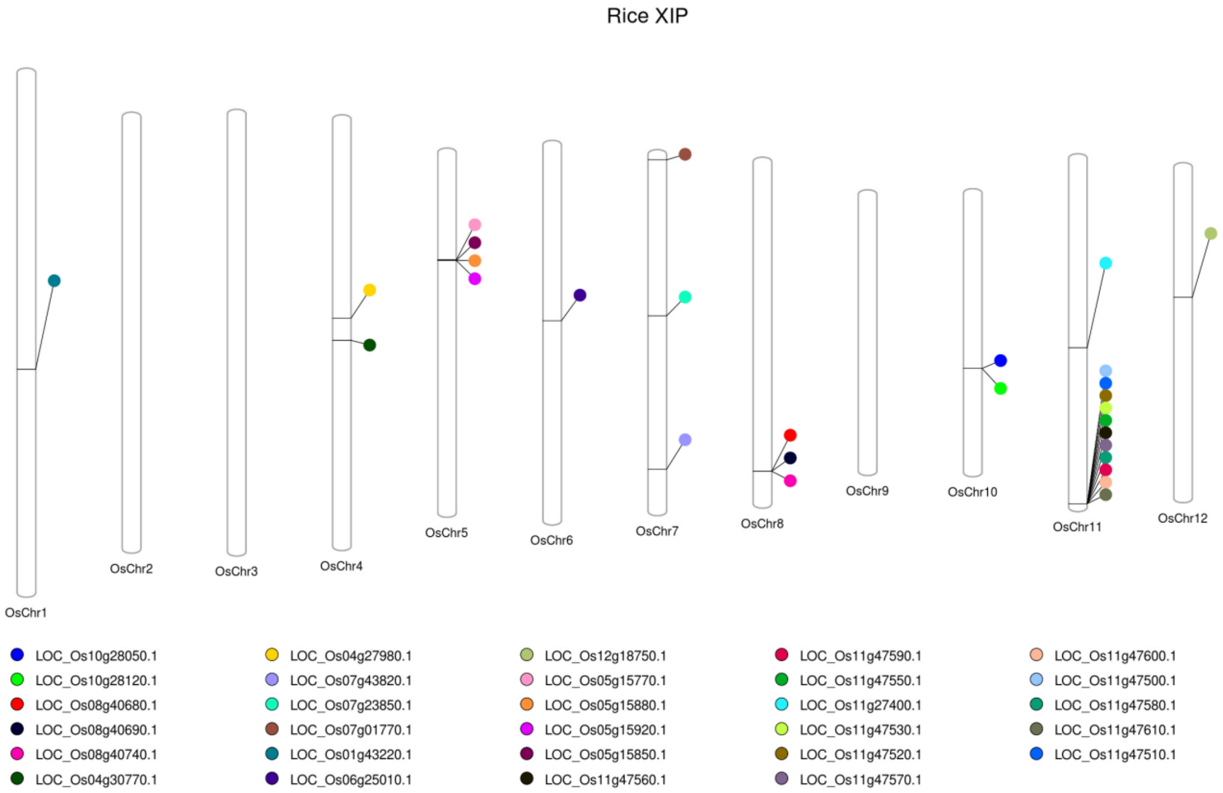

Figure S9: Schematic representation of the genomic organization of XIP family members in the rice genome. OsChr, chromosome.

A)

Organism	Blast Name	Score	Number of Hits	Description
commelinids	monocots		91	
. Poaceae	monocots		90	
. . Pooideae	monocots		76	
. . . Triticeae	monocots		74	
. . . . Triticinae	monocots		59	
. . . . . Triticum	monocots		53	
. . . . . . Triticum aestivum	monocots	2398	36	Triticum aestivum hits
. . . . . . Triticum dicoccoides	monocots	2350	8	Triticum dicoccoides hits
. . . . . . Triticum urartu	monocots	2056	8	Triticum urartu hits
. . . . . . Triticum turgidum subsp. durum	monocots	1674	1	Triticum turgidum subsp. durum hits
. . . . . Aegilops tauschii subsp. strangulata	monocots	1940	6	Aegilops tauschii subsp. strangulata hit
. . . . Secale cereale	monocots	1688	3	Secale cereale hits
. . . . Hordeum vulgare subsp. vulgare	monocots	1574	10	Hordeum vulgare subsp. vulgare hits
. . . . Hordeum vulgare	monocots	1487	2	Hordeum vulgare hits
. . . Lolium rigidum	monocots	647	2	Lolium rigidum hits
. . Sorghum bicolor	monocots	252	1	Sorghum bicolor hits
. . Setaria viridis	monocots	246	3	Setaria viridis hits
. . Setaria italica	monocots	241	2	Setaria italica hits
. . Panicum hallii	monocots	102	1	Panicum hallii hits
. . Panicum virgatum	monocots	99.0	1	Panicum virgatum hits
. . Digitaria exilis	monocots	91.6	6	Digitaria exilis hits
. Musa acuminata	monocots	327	1	Musa acuminata hits

B)

Organism	Blast Name	Score	Number of Hits	Description
Eukaryota	eukaryotes		154	
. Poaceae	monocots		153	
. . BOP clade	monocots		77	
. . . Pooideae	monocots		56	
. . . . Triticeae	monocots		49	
. . . . . Triticinae	monocots		40	
. . . . . . Triticum	monocots		38	
. . . . Triticum aestivum	monocots	570	24	Triticum aestivum hits
. . . . Triticum turgidum subsp. durum	monocots	560	6	Triticum turgidum subsp. durum hits
. . . . Triticum urartu	monocots	530	4	Triticum urartu hits
. . . . Triticum dicoccoides	monocots	518	4	Triticum dicoccoides hits
. . . . . . Aegilops tauschii subsp. strangulata	monocots	567	2	Aegilops tauschii subsp. strangulata h
. . . . . Hordeum vulgare subsp. vulgare	monocots	545	5	Hordeum vulgare subsp. vulgare hits
. . . . . Hordeum vulgare	monocots	545	4	Hordeum vulgare hits
. . . . Lolium rigidum	monocots	485	5	Lolium rigidum hits
. . . . Brachypodium distachyon	monocots	457	2	Brachypodium distachyon hits
. . . Oryza meyeriana var. granulata	monocots	441	3	Oryza meyeriana var. granulata hits
. . . Oryza sativa Japonica Group	monocots	429	12	Oryza sativa Japonica Group hits
. . . Oryza brachyantha	monocots	418	3	Oryza brachyantha hits
. . . Zizania palustris	monocots	397	1	Zizania palustris hits
. . . Oryza sativa	monocots	325	2	Oryza sativa hits
. . Miscanthus lutarioriparius	monocots	431	1	Miscanthus lutarioriparius hits
. . Eleusine coracana subsp. coracana	monocots	431	10	Eleusine coracana subsp. coracana h
. . Panicum hallii var. hallii	monocots	428	2	Panicum hallii var. hallii hits
. . Panicum hallii	monocots	427	3	Panicum hallii hits
. . Panicum virgatum	monocots	427	7	Panicum virgatum hits
. . Panicum miliaceum	monocots	426	5	Panicum miliaceum hits
. . Setaria italica	monocots	425	10	Setaria italica hits
. . Setaria viridis	monocots	425	10	Setaria viridis hits
. . Sorghum bicolor	monocots	424	9	Sorghum bicolor hits
. . Zea mays	monocots	422	4	Zea mays hits
. . Digitaria exilis	monocots	417	9	Digitaria exilis hits
. . Eragrostis curvula	monocots	411	5	Eragrostis curvula hits
. . Dichanthelium oligosanthes	monocots	339	1	Dichanthelium oligosanthes hits
. Artibeus jamaicensis	bats	561	1	Artibeus jamaicensis hits

Figure S10: Basic Local Alignment Search Tool (BLAST) search results using (A) TAXI-I (AJ438880.1) and (B) XIP-I (ADB81849.1) as query on National Center for Biotechnology Information (NCBI).
